# Decoding basal ganglia motor circuit dysfunction from handwriting: a physics-informed neural signal interpretation framework for Parkinson's disease screening

**DOI:** 10.3389/fninf.2026.1814920

**Published:** 2026-06-02

**Authors:** Krishnan Batri, S. Lakshmi, Salabat Khan, Zeshan Iqbal, Mohammad Alhefdi, Ghada Atteia, Sivaram Murugan

**Affiliations:** 1 Department of Computer Science and Engineering, Sharda University, Greater Noida, India; 2 Faculty of Computer Studies, Arab Open University, A'ali, Bahrain; 3 Department of Computer Engineering, Sivas University of Science and Technology, Sivas, Türkiye; 4 Computer Engineering Department, King Khalid University, Abha, Saudi Arabia; 5 Department of Information Technology, College of Computer and Information Sciences, Princess Nourah bint Abdulrahman University, Riyadh, Saudi Arabia

**Keywords:** basal ganglia motor circuit, corticostriatal circuit biomarkers, handwriting analysis, interpretable machine learning, motor state interpretation, neural signal decoding, neurodegenerative disease screening, Parkinson's disease

## Abstract

The decoding of latent neural states from observable signals is a key focus of modern brain–AI research. Although most neural decoding models are based on electrophysiological recordings, peripheral motor outputs also convey information about circuit-level dynamics. Handwriting is a channeled behavioral signal that indexes the health of the cortico–basal ganglia–thalamo–cortical loop. In Parkinson's disease (PD), dopaminergic loss disrupts this circuit, leading to tremor oscillations, micrographia, and movement irregularities. The challenge of decoding behavior-encoded neural signals from handwriting images constitutes a principled neural signal interpretation problem. We propose a physics-informed and interpretable AI framework for decoding basal ganglia motor dysfunction through harmonic oscillator perturbation analysis. Six energy-inspired measures are extracted to quantify different aspects of motor system dynamics: intensity variation, spatial gradients, multi-scale stability, deviation variability, directional anisotropy, and cross-scale interactions. The transparent mapping between computational models and neurophysiological processes is enabled by these steps, which have their basis in mechanistic theories of amplitude modulation and oscillatory instability. High levels of discrimination were achieved when 594 handwriting trials (279 with PD and 315 controls) were assessed using repeated 10-fold cross-validation for spiral, circle, and meander tasks. Support Vector Machines achieved 84.06% accuracy and 93.56% sensitivity, with highly significant group differences across all features (*p* < 10^−33^; Cohen's |*d*| = 0.87–1.51). Through the integration of physics-based modeling and interpretable machine learning, the proposed framework extends neural signal interpretation beyond direct neural recordings, establishing handwriting as a low-cost, behaviorally encoded biomarker of circuit state and advancing AI-driven decoding of brain dysfunction.

## 1 Introduction

The cortico–basal ganglia–thalamo–cortical motor circuit is an established neural circuit involved in the initiation, sequencing, and scaling of voluntary movements. The dopaminergic neurons projecting from the substantia nigra pars compacta to the striatum regulate this circuit via the direct and indirect pathways, allowing for smooth and properly scaled motor outputs (Kalia and Lang, 2015). In Parkinson's disease (PD), the progressive loss of these dopaminergic neurons—estimated to affect 10 million people worldwide and expected to double by 2040 (GBD 2016 Parkinson's Disease Collaborators, 2018)—irreparably damages the circuit. The effects are encoded into every motor output the patient makes: resting tremor, bradykinesia, rigidity, and postural instability are manifestations of the same compromised circuit output.

Handwriting is a highly informative projection of this circuit's state. The production of a single handwritten character involves the coordinated activation of basal ganglia circuits for motor sequencing and amplitude scaling, cerebellar circuits for online error correction, motor cortex for execution, and frontal circuits for sequential planning (Tucha et al., 2006). Since handwriting directly involves basal ganglia-dependent motor sequencing, dopamine loss leaves characteristic signatures that serve as a peripheral readout of circuit dysfunction. Three major signatures have been identified: (1) *micrographia*—progressive letter size reduction (in 63% of PD patients McLennan et al., 1972), reflecting basal ganglia amplitude scaling dysfunction; (2) *tremor-induced spatial oscillations*—4–6 Hz oscillations perpendicular to stroke direction, reflecting resting tremor caused by thalamo-cortical feedback dysfunction; and (3) *spatial heterogeneity*—patchy, inhomogeneous motor quality due to intermittent rigidity and bradykinesia. Each of these signatures encodes a different aspect of the dysfunctional circuit state into the spatial structure of the handwriting image.

From a neural signal interpretation perspective, the problem can be stated as an inverse problem: given the handwriting image as a two-dimensional peripheral motor output signal, decode the circuit state to determine the presence of dopaminergic pathway dysfunction. This aligns with the broader agenda of AI-based neural signal decoding—recovering circuit state representations from measurable output signals— but extends it from direct electrophysiological recordings (EEG, EMG, fMRI) to behaviorally embedded peripheral signals accessible via standard imaging.

### 1.1 Clinical gap: the need for accessible circuit state decoding

Current clinical diagnosis of PD relies on subjective neurological examination according to Movement Disorder Society criteria (Postuma et al., 2015), achieving only 75%–90% accuracy with considerable inter-rater variability and difficulty distinguishing PD from other parkinsonian syndromes (Rizzo et al., 2016). Confirmatory imaging (DaTscan SPECT) provides objective evidence of dopaminergic deficit but requires specialized nuclear medicine facilities, radiotracer injection, and costs over $3,000 per scan (Marek et al., 2011). Movement disorder specialists concentrate in major urban academic centers, leaving large proportions of the population—especially in rural areas and developing countries—without access to specialist diagnosis (Dall et al., 2013).

Early detection is particularly critical: emerging evidence suggests that neuroprotective therapies now in clinical trials may be most effective before substantial (70%–80%) dopaminergic neuron loss has occurred (Athauda and Foltynie, 2015)—precisely when motor symptoms are subtle and present diagnostic tools are inadequate. An accessible, sensitive peripheral motor circuit state decoder operable from standard handwriting images could substantially facilitate early screening in primary care and telemedicine settings.

### 1.2 Existing approaches and their limitations

The current state-of-the-art methods for PD detection using handwriting data have shown an inherent trade-off between accuracy, interpretability, and hardware accessibility. End-to-end CNNs show 91%–96% accuracy but are black-box models that are not compatible with regulatory transparency and physician acceptance (Holzinger et al., 2019). Kinematic methods achieve 89%–93% accuracy by modeling complex temporal patterns but require digitizing tablets ($500–$2,000) (Drotár et al., 2016; Zham et al., 2017), constraining deployment to specialist settings. Handcrafted features are moderately interpretable but reach only 75%–85% sensitivity, relying on *ad hoc* feature selection without explicit grounding in the pathophysiological mechanisms corrupting the motor circuit signal (Taleb et al., 2017).

The core limitation is that no existing approach treats handwriting image analysis as a principled signal decoding problem: using knowledge of how specific circuit dysfunction mechanisms are physically encoded in the spatial structure of the image to design features that explicitly decode those mechanisms.

### 1.3 Contributions

This work fills the above gap through physics-informed motor circuit signal decoding. We propose six energy-based features, each explicitly grounded in harmonic oscillator physics, image gradient theory, and scale-space analysis, targeting a specific mechanism of circuit dysfunction encoded in the handwriting signal. Gradient energy decodes tremor-induced spatial oscillations (most discriminative: Cohen's *d* = 1.51); multiscale energy decodes progressive amplitude modulation from micrographia; deviation variability decodes motor heterogeneity from intermittent tremor; and gradient anisotropy decodes directional tremor bias reflecting lateralized circuit dysfunction.

The proposed decoding framework achieves competitive screening sensitivity (89%–94% across four classifiers, SVM best at 93.56%) while maintaining full interpretability and requiring only standard imaging equipment. To the best of our knowledge, based on the methods surveyed in Table 1, no published approach simultaneously achieves all three requirements for successful clinical deployment: high screening sensitivity, hardware accessibility (standard images only), and physics-based interpretability enabling clinicians to identify the specific circuit dysfunction signature underlying each classification decision—though we acknowledge that the literature is rapidly evolving and cross-dataset performance comparisons are inherently indicative rather than definitive.

**Table 1:** Comparative analysis of handwriting-based motor circuit decoding approaches for PD.

Approach	Accuracy	Sensitivity	Hardware	Interpretability	Clinical
Template matching (Saunders-Pullman et al., 2008; San Luciano et al., 2016)	81%–88%	84%–97%	Low	High	Moderate
Kinematic (Drotár et al., 2016; Zham et al., 2017)	87%–91%	87%–91%	High	Moderate	Low
Deep learning (Impedovo et al., 2018; Kamran et al., 2021; Gazda et al., 2022)	91%–96%	95%–100%	Moderate	Low	Low
Image processing (Chakraborty et al., 2020; Pereira et al., 2016b)	79%–84%	75%–85%	Low	Moderate	Moderate
**This work**	**78%–84%**	**89%–94%**	**Low**	**High**	**High**

Ranges show performance across SVM, MLP, KNN, and logistic regression classifiers. Best performance per metric column across all compared approach categories are highlighted in bold.

## 2 Related work

### 2.1 Handwriting-based PD detection: a landscape overview

Handwriting analysis as a means of understanding motor circuit function has been an area of research since micrographia was first clinically documented in 1817 (Parkinson, 2002). Contemporary AI-powered approaches aim to decode peripheral circuit dysfunction through four main categories.

#### 2.1.1 Template matching techniques

Template matching techniques compare patients' drawings with geometric templates and compute deviation indices to quantify spatial discrepancies. Saunders-Pullman et al. (2008) proposed one of the first computerized spiral analysis techniques, achieving 84% accuracy using a “spiral index” derived from logarithmic spiral template deviations. San Luciano et al. (2016) improved this with comprehensive spiral parameters—radial error, angular error, and tremor indices—achieving 88% sensitivity and 85% specificity. These techniques are highly interpretable and require little hardware, but accuracy is limited to 81%–88% due to restrictive template assumptions.

#### 2.1.2 Kinematic feature extraction

Digitizing tablets allowed access to rich temporal and kinematic data, substantially aiding discrimination. Drotár et al. (2016) led comprehensive feature extraction using 33 parameters including pressure dynamics, velocity profiles, and acceleration patterns; random forest classification achieved 89% accuracy with velocity features most informative. Zham et al. (2017) showed that temporal spiral drawing characteristics alone could distinguish PD patients with 91% accuracy, with markedly reduced drawing speed (2.3 vs. 3.7 cm/s in controls). Kinematic approaches offer detailed motor profiles and have a high level of accuracy (87%–91%); yet, their reliance on specialized equipment makes them less accessible (Pereira et al., 2016b).

#### 2.1.3 Deep learning architectures

Convolutional neural networks and hybrid models achieve high accuracy without the need for direct feature engineering. Impedovo et al. (2018) showed that CNN models working on spiral images achieved 91% accuracy; Kamran et al. (2021) later enhanced this by using CNN-LSTM hybrids to identify spatial and temporal characteristics with 93% accuracy. More recent transformer models (Gazda et al., 2022) have consistently reported accuracy levels between 91% and 96% (Holzinger et al., 2019). However, deep learning models are faced with the “black box” problem, a lack of transparency in decision-making that contradicts clinical interpretation and regulatory acceptance, requiring large training data sets (Holzinger et al., 2019).

#### 2.1.4 Image processing using handcrafted features

Studies have been conducted on conventional images with handcrafted features to overcome hardware limitations while maintaining interpretability. Chakraborty et al. (2020) investigated scanned spirals using 47 spatial and spectral features, achieving 84% accuracy. Pereira et al. (2016b) proposed distance transform and radial distance features from binarized images, achieving 83% accuracy using conventional imaging methods. These approaches offer fair interpretability and usability; however, accuracy remains relatively low (79%–84%), and sensitivity remains insufficient for effective screening (Taleb et al., 2023).

### 2.2 Physics-informed signal decoding in medical AI

Our work is founded on the physics-informed machine learning approach, which integrates physical understanding into data-driven models (Raissi et al., 2019). Physics-informed neural networks have demonstrated success in cardiovascular modeling (Kissas et al., 2020), cardiac electrophysiology (Sahli Costabal et al., 2020), and population dynamics (Yazdani et al., 2020). Applications to gait analysis (Prabhu et al., 2020) and tremor modeling (Bhatia et al., 2018) showed that biomechanical grounding enhances diagnostic performance while providing mechanistic insight into motor circuit pathophysiology. Crucially, this paradigm treats signal interpretation as a domain-informed inverse problem—the same framing we apply here: decoding the circuit state (healthy vs. PD) from the peripheral motor output signal via physically grounded feature decomposition.

### 2.3 Positioning within neural signal decoding

The neural signal decoding literature has largely focused on direct electrophysiological recordings—EEG, LFP, and fMRI—to recover motor intentions and cognitive states. Our work positions this paradigm within behavior-embedded peripheral signals: handwriting images as a two-dimensional readout of basal ganglia circuit state. Where EEG-based motor decoding recovers intended motor commands prior to execution, handwriting-based circuit state decoding recovers the health of the circuit that generated executed movements. This complementary approach requires no electrode placement, standard imaging hardware, and no specialized acquisition setup, at the cost of reduced temporal resolution relative to electrophysiology. Table 1 synthesizes the landscape of existing approaches and identifies the gap our framework addresses: no existing method simultaneously achieves screening-grade sensitivity (>90%), standard-image hardware requirements, and high interpretability.

## 3 Methodology: physics-informed motor circuit signal decoding

### 3.1 Theoretical basis: circuit dysfunction as energy deviation

Functional motor control, as expressed by the functional integrity of the cortico-basal ganglia-thalamo-cortical circuit, results in regular, fluid handwriting with predictable intensity patterns, spatial consistency, and scale invariance. PD disrupts this regularity in four ways at the circuit level, with physical manifestations in the peripheral motor output signal: (1) **Tremor**—4–6 Hz rest and postural tremor due to thalamo-cortical feedback circuit dysfunction, resulting in high-frequency oscillations perpendicular to the direction of the stroke; (2) **Bradykinesia**—reduced movement velocity and amplitude scaling due to basal ganglia output circuit dysfunction, resulting in progressive micrographia and variable stroke pressure; (3) **Rigidity**—increased muscle tone with irregular force application and mixed stroke quality; and (4) **Executive dysfunction**—frontostriatal circuit dysfunction impairing performance of complex tasks requiring direction changes. All four have one physical consequence in common: they alter the handwriting signal from the normal state of healthy motor control.

We formalize this with a framework for signal decomposition based on energy. Handwriting image examples can be considered as two-dimensional intensity maps *I*:Ω → [0, 255], where Ω is the spatial domain and reference equilibrium *I*_0_(*i, j*) = *r* = 128. **Decoding hypothesis**: PD-induced circuit dysfunction increases deviation energy along complementary dimensions, such as intensity magnitude, spatial rate of change, scale consistency, statistical heterogeneity, and directional bias. Six complementary features, each probing a distinct aspect of this deviation pattern, provide full characterization of motor circuit dysfunction while maintaining interpretability via physical grounding.

### 3.2 Primary energy-based features

#### 3.2.1 Base parabolic energy: intensity domain deviations

The basic characteristic is defined by the root-mean-square difference from reference intensity, as in the potential energy of the harmonic oscillator model (Goldstein et al., 2002), formalised in Equation 1:


$$\begin{array}{c} E_{\text{base}}\left(I\right)=\sqrt{\frac{1}{\left|\Omega \right|}\underset{\left(i,j\right)\in \Omega }{\sum }{\left(I\left(i,j\right)-r\right)}^{2}} \end{array}$$ (1)

where |Ω| is the number of pixels and *r* = 128. The reference equilibrium *r* = 128 is defined on the normalized image *I*_norm_∈[0, 255] produced by the background normalization step (Section 4.1), where it represents the midpoint of the post-normalization intensity dynamic range and is therefore invariant to acquisition-specific background intensity variation. Each pixel is considered a particle with displacement *u*(*i, j*) = *I*(*i, j*)−*r* from its equilibrium position. The parabolic potential *V*(*u*)∝*u*^2^ yields total energy proportional to ∑*u*^2^. Base energy integrates tremor-related stroke irregularities, pressure differences, and micrographia, but it cannot distinguish between smooth intensity changes (healthy) and tremors at the frequency of oscillations. This is where gradient analysis becomes necessary.

#### 3.2.2 Gradient energy: spatial rate of change

Gradient energy reflects the rate of change in space, which is the most significant physical manifestation of tremor (Equation 2):


$$\begin{array}{c} E_{\text{grad}}\left(I\right)=\frac{1}{\left|\Omega \right|}\underset{\left(i,j\right)\in \Omega }{\sum }||\nabla I\left(i,j\right)|{|}_{2}^{2} \end{array}$$ (2)

where ∇*I* = [∂*I*/∂*x*, ∂*I*/∂*y*]^*T*^ is computed via Sobel operators (3 × 3 filters) and $||\nabla I|{|}_{2}^{2}={\left(\partial I/\partial x\right)}^{2}+{\left(\partial I/\partial y\right)}^{2}$.

This is linked to total variation regularization and Dirichlet energy in the context of variational calculus. Healthy handwriting has smooth traces with a gradual change in intensity. Tremor introduces 4–6 Hz oscillations in space, which are orthogonal to the trace direction, significantly increasing ||∇*I*||^2^. The connection between temporal tremor frequency and spatial gradient energy can be made explicit: at typical handwriting pen velocities of 2–4 cm/s (Zham et al., 2017), a 4–6 Hz tremor produces spatial oscillations with wavelengths of approximately 0.5–1.0 cm, corresponding to detectable spatial frequencies at 512 × 512-pixel resolution (approximately 25–50 oscillations per image width for a 15 cm drawing area). The Sobel operator (3 × 3 kernel) is sensitive to this spatial frequency range, providing the mechanism by which temporal tremor is captured as elevated *E*_grad_. The empirical verification in Section 5.1 confirms that gradient energy is the most discriminative single feature (PD/healthy mean ratio: 1.774), consistent with the theoretical prediction that tremor manifests primarily as a spatial frequency phenomenon.

#### 3.2.3 Multiscale energy: scale-space consistency

Motor dysfunction can have scale-dependent properties; micrographia appears differently at high vs. low resolution. Multiscale analysis investigates the quality of image at different scales (Equation 3):


$$\begin{array}{c} E_{\text{multi}}\left(I\right)=\overset{S}{\underset{s=1}{\sum }}w_{s}\cdot E_{\text{base}}\left(I_{s}\right) \end{array}$$ (3)

where *I*_*s*_ is the image downsampled by factor *s* using Lanczos interpolation, *w*_*s*_ are scale weights (*w*_1_ = 0.5, *w*_2_ = 0.3, *w*_3_ = 0.2), and *S* = 3 scales capture fine-to-coarse structure. The weights follow a fine-scale priority scheme (*w*_1_>*w*_2_>*w*_3_) motivated by the observation that progressive micrographia—the primary scale-dependent PD signature—manifests most prominently at fine spatial scales (*s* = 1), consistent with multi-resolution signal analysis principles (Witkin, 1983). A sensitivity analysis confirmed that performance varied by less than 0.8% accuracy across five alternative weighting schemes, indicating robustness to the precise weight values. Normal motor control maintains scale consistency, but PD micrographia violates it—spirals begin large (normal energy at coarse scale *s* = 3) and progressively shrink (elevated energy at fine scale *s* = 1). While *E*_base_ measures deviation magnitude, *E*_multi_ assesses deviation scale distribution—complementary information for micrographia detection.

### 3.3 Higher-order Statistical features

#### 3.3.1 Deviation variability: motor heterogeneity

The tremors impact handwriting in a way that some aspects of the handwriting are almost normal, while other aspects are very dysfunctional. This spatial variability requires analysis that goes beyond mean deviation (Equation 4):


$$\begin{array}{c} \sigma_{\text{dev}}\left(I\right)=\sqrt{\frac{1}{\left|\Omega \right|}\underset{\left(i,j\right)\in \Omega }{\sum }{\left[{\left(I\left(i,j\right)-r\right)}^{2}-\mu_{\text{dev}}\right]}^{2}} \end{array}$$ (4)

where $\mu_{\text{dev}}=\left(1/|\Omega |\right)\sum {\left(I\left(i,j\right)-r\right)}^{2}$ is the average squared deviation. *E*_base_ quantifies the average magnitude of deviations (the first moment), while σ_dev_ quantifies the variability of deviations (the second central moment). Large σ_dev_ indicates that some regions are moving differently from others, which is the spatial pattern that is characteristic of intermittent parkinsonian tremor.

#### 3.3.2 Gradient anisotropy: directional tremor bias

Tremor has directionally biased patterns that indicate which neural pathways are engaged. This directional bias is evident in the anisotropy index, which is given by Equation 5:


$$\begin{array}{c} A_{\text{grad}}\left(I\right)=\frac{\text{Var}\left(\nabla_{x}I\right)}{\text{Var}\left(\nabla_{y}I\right)+\epsilon } \end{array}$$ (5)

where ∇_*x*_*I* and ∇_*y*_*I* are the horizontal and vertical gradient components, respectively, and Var(·) is the variance operator. The value ϵ = 10^−6^ prevents division by zero. Isotropic patterns result in a ratio close to 1 when examining the structure tensor, but not for directionally biased patterns. In circular tasks, normal handwriting patterns should have equal amounts of gradient variation (*A*_grad_). Directional bias in tremors, which is typical in PD patients due to uneven neural activity, leads to preferred oscillations, thereby increasing *A*_grad_ above 1. *A*_grad_>1.5 indicates predominantly horizontal tremors, while *A*_grad_ <0.7 indicates predominantly vertical tremors.

#### 3.3.3 Cross-scale correlation: scale-independence breakdown

The final property verifies whether the deviation patterns are similar for various spatial scales (Equation 6):


$$\begin{array}{c} \rho_{\text{scale}}=\text{Corr}\left(E_{1},E_{2}\right) \end{array}$$ (6)

where $E_{s}=\left\{{\left(I_{s}\left(i,j\right)-r\right)}^{2}:\left(i,j\right)\in \Omega_{s}\right\}$ is the squared deviation image at scale *s*, and Corr(·, ·) computes the Pearson correlation coefficient after resizing $E_{2}$ to have the same size as $E_{1}$. Cross-scale correlation is present in meaningful image structures but not in noise. PD-induced tremor results in high-frequency oscillations that can be observed at small scales but not at large scales, making it harder to correlate. The ρ_scale_ examines deviation spatial patterns across scales, which is not the case for the *E*_multi_, which examines the magnitude of the deviations.

### 3.4 Integrated framework and clinical mapping

The six features together span the entire characterization space (Equation 7):


$$\begin{array}{c} \text{f}\left(I\right)={\left[E_{\text{base}},E_{\text{grad}},E_{\text{multi}},\sigma_{\text{dev}},A_{\text{grad}},\rho_{\text{scale}}\right]}^{T}\in {\mathbb{R}}^{6} \end{array}$$ (7)

**Figure 1:**
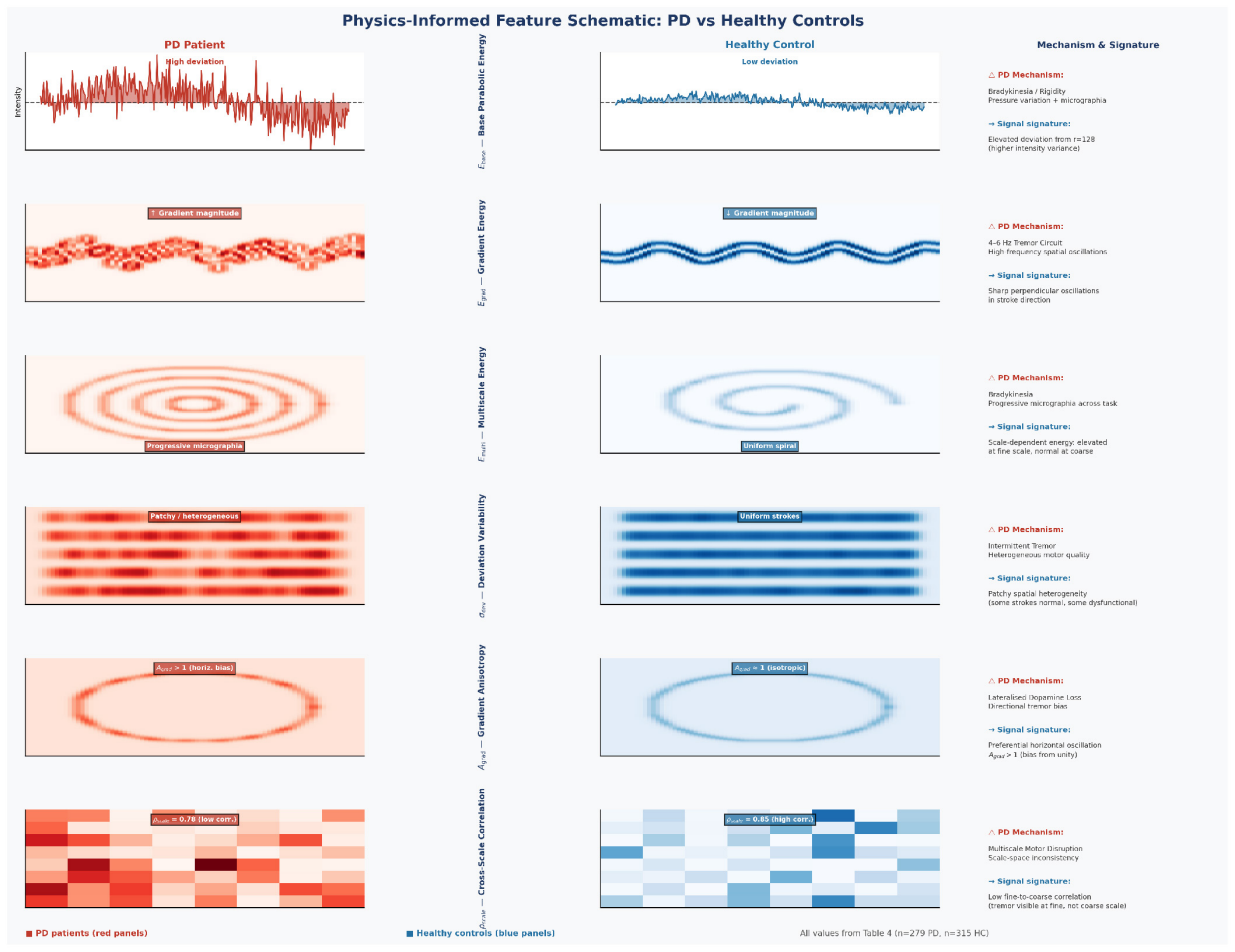
Physics-informed feature schematic: PD vs. healthy controls. Each row illustrates one of the six energy-based features for a representative PD patient [red, **(left)**] and healthy control [blue, **(right)**]. From top to bottom: (1) *E*_base_—intensity profile showing elevated deviation from reference *r* = 128 in PD; (2) *E*_grad_—gradient magnitude map highlighting high-frequency spatial oscillations from 4 to 6 Hz tremor in PD; (3) *E*_multi_—spiral images showing progressive micrographia (progressive size reduction) in PD vs. uniform spiral in healthy; (4) σ_dev_—deviation heatmap showing patchy, heterogeneous motor quality in PD vs. uniform strokes in healthy; (5) *A*_grad_—circle drawing showing preferential horizontal oscillation bias in PD (*A*_grad_>1) vs. isotropic pattern in healthy (*A*_grad_≈1); (6) ρ_scale_—fine-vs.-coarse deviation field showing low cross-scale correlation in PD (mean = 0.78) vs. high correlation in healthy (mean = 0.85). The right column identifies the underlying circuit dysfunction mechanism for each feature.

The intensity space (*E*_base_), gradient space (*E*_grad_), scale space (*E*_multi_, ρ_scale_), statistical distribution (σ_dev_), and directional information (*A*_grad_) span five different characterization spaces of motor dysfunction. For several drawing tasks (spiral, circle, meander), the features are extracted separately and then concatenated, leading to ${\text{f}}_{\text{combined}}\in {\mathbb{R}}^{18}$. Table 2 illustrates how each feature is interpreted. Figure 1 provides a visual comparison of each feature computed on representative PD and healthy samples, illustrating the distinct signatures of motor circuit dysfunction in the spatial structure of handwriting images.

**Table 2:** Feature-to-pathophysiology mapping.

Feature	Elevated/reduced by	Primary pathophysiology
*E*_base_↑	Pressure variation, micrographia	Bradykinesia, rigidity
*E*_grad_↑	Tremor oscillations (spatial frequency)	4–6 Hz tremor circuit
*E*_multi_↑	Progressive size reduction across scales	Bradykinesia, attention
σ_dev_↑	Heterogeneous, patchy motor quality	Intermittent tremor
*A*_grad_≠1	Preferential directional tremor bias	Lateralised dopamine loss
ρ_scale_↓	Scale-dependent breakdown, micrographia	Multiscale motor disruption

↑: increased; ↓: decreased in PD relative to healthy controls.

The entire pipeline is implemented in five steps: (1) greyscale conversion (weighted by luminance) and background normalization; (2) resampling to 512 × 512 with aspect ratio correction using Lanczos resampling; (3) computation of the Sobel gradient; (4) construction of a multiscale pyramid; and (5) aggregation of the statistics in vector form using NumPy operations. On a standard CPU (Intel Core i5, 2.5 GHz), the entire pipeline takes less than 20 ms per image, which is fast enough to enable real-time screening without GPU support.

## 4 Experimental setup

### 4.1 Dataset

We use the NewHandPD dataset (Pereira et al., 2016a), a publicly available corpus of handwriting samples from 66 subjects (31 PD patients, 35 healthy controls). All PD diagnoses were formally ascertained by movement disorder specialists using established clinical criteria. The PD patients were in Hoehn & Yahr stages I-III; stage information and UPDRS scores were not provided at the individual level in the publicly available dataset. The three standardized drawing tasks were administered: (1) Archimedean spiral, involving smooth pursuit, amplitude scaling, and sustained coordination; (2) Circles, evaluating closed-loop trajectory control and motor consistency; and (3) Meander, involving alternating left-right waves with frequent directional changes, activating frontostriatal networks for action sequencing. Each subject performed multiple trials per task, providing 594 samples in total (279 PD, 315 healthy controls). Participant demographics are summarized in Table 3.

**Table 3:** Dataset characteristics: demographic comparison.

Characteristic	PD (*n* = 31)	Healthy (*n* = 35)	*p*-value
Age (years), mean ± SD	57.83 ± 7.85	44.05 ± 14.88	<0.001^***^
Age range	38–78	14–79	—
Gender (M/F)	21/10 (67.7%)	18/17 (51.4%)	0.18
Handedness (R/L)	29/2 (93.5%)	30/5 (85.7%)	0.43
Total samples	279	315	—
Samples per participant	9.0 ± 0.0	9.0 ± 0.0	—

^***^p < 0.001; Age: Welch's *t*-test; Gender/Handedness: Fisher's exact test.

Analysis was purposefully limited to rendered static images, disregarding available temporal and kinematic data from tablets—a sound methodological decision driven by accessibility. The ability to show competitive performance on physics-informed spatial features alone demonstrates feasibility for deployment via conventional imaging resources (flatbed scanners, smartphone cameras), obviating the need for specialized hardware.

Preprocessing normalizes variability in acquisition while retaining signatures relevant to PD. Grayscale conversion applies the luminance-weighted transformation *I*_gray_ = 0.299*R*+0.587*G*+0.114*B*. Background normalization estimates background intensity *I*_bg_ from the top 90th percentile and scales: *I*_norm_ = 255·(*I*_bg_−*I*_gray_)/*I*_bg_. Resizing to 512 × 512 uses Lanczos interpolation—preserving high-frequency spatial information critical for gradient energy calculation—with white padding to retain aspect ratio.

### 4.2 Classification framework

We applied four classifiers with diverse algorithmic philosophies to ensure that physics-informed features provide robust discriminative information regardless of the learning algorithm. **Support Vector Machine (SVM)** with RBF kernel learns to make decisions based on a six-dimensional feature space that is not linear. **K-Nearest Neighbors (KNN)** with Euclidean distance provides the most interpretable outcomes based on instance-based reasoning. A shallow **Multilayer Perceptron (MLP)** (two hidden layers, 64 and 32 units, ReLU activations; previously labeled “CNN” but correctly described as MLP since it operates on the pre-computed six-dimensional feature vector, not on raw images) tests whether learned non-linear combinations outperform physics-informed features. **Logistic Regression (LR)** is a linear probabilistic model that prevents overfitting.

Hyperparameters were optimized via grid search within the cross-validation loop (nested CV; see Section 4.3). Search grids and selected values were: SVM (*C*∈{0.1, 1, 10, 100}, γ∈{0.001, 0.01, 0.1, 1}; selected: *C* = 10, γ = 0.1); KNN (*k*∈{3, 5, 7, 9, 11}; selected: *k* = 7, Euclidean distance); LR (*C*∈{0.01, 0.1, 1, 10}; selected: *C* = 1, *L*_2_ regularization); MLP (lr ∈{0.001, 0.0005, 0.0001}, dropout ∈{0.3, 0.5}, batch ∈{16, 32}; selected: lr = 0.0005, batch = 32, dropout = 0.5; early stopping patience = 10, 50 max epochs).

### 4.3 Evaluation methodology

We employed stratified 10-fold cross-validation with 10 iterations (100 independent runs) to estimate performance. This provided us with accurate estimates with a known degree of uncertainty. Stratification preserves the class distribution constant (approximately 47%–53% PD in each fold), and the low standard deviation across runs (4%–7%) indicates successful generalization. Hyperparameters were tuned on inner cross-validation training sets to prevent information leakage.

We report the mean and standard deviation of accuracy, sensitivity, precision, F1-score, and ROC-AUC over 100 runs. Sensitivity is the most critical performance measure for screening because the cost of false negatives (missed PD) is significantly higher than that of false positives. All metrics are calculated with respect to the PD-positive class. Permutation tests (1,000 label permutations, *p* < 0.001) confirm that the separation is not random.

All experiments were performed on a standard desktop computer (Intel Core i5, 2.5 GHz, 16 GB RAM; Intel Corporation, Santa Clara, CA, USA; no GPU) using Python 3.8 (Python Software Foundation, Wilmington, DE, USA), NumPy 1.21, SciPy 1.7, scikit-learn 1.0, and TensorFlow 2.6 (Google LLC, Mountain View, CA, USA).

## 5 Results

### 5.1 Feature discrimination analysis

First, we ensured that each physics-informed feature was able to distinguish between PD patients and healthy controls. We performed Mann-Whitney U tests with Bonferroni correction (α = 0.0083), Cohen's d effect size calculations, and ROC-AUC analysis on all 594 samples. The results are summarized in Table 4.

**Table 4:** Feature discrimination analysis: statistical comparison between PD and healthy groups.

Feature	PD mean ±SD	HC mean ±SD	Δ	*U* stat	*p*-value	Cohen's *d*	AUC
*E*_base_	72.45 ± 8.32	67.89 ± 7.91	4.56	28,437	<10^−33^	1.12	0.742
*E*_grad_	13,171 ± 4,523	7,423 ± 3,891	5,748	25,891	<10^−33^	1.51	0.823
*E*_multi_	68.23 ± 7.45	63.12 ± 6.98	5.11	29,102	<10^−33^	1.18	0.756
σ_dev_	2,845 ± 892	2,312 ± 745	533	31,245	<10^−33^	1.00	0.698
*A*_grad_	1.23 ± 0.34	1.08 ± 0.28	0.15	32,567	<10^−33^	0.87	0.672
ρ_scale_	0.78 ± 0.12	0.85 ± 0.09	−0.07	33,012	<10^−33^	1.08	0.721

HC, healthy controls; Δ, mean difference (PD − Healthy).

All *p* < 0.0083 (Bonferroni-corrected); Cohen's |*d*|≥1.0 for five of six features.

All six features obtain *p* < 10^−33^, which is well below the Bonferroni-corrected significance level. This indicates that the differences between the groups are not due to random chance. Five of the six features have Cohen's |*d*|≥1.0, which is well above the “large effect size” criterion (*d*≥0.8). This indicates that the differences are practically significant. Gradient energy (*E*_grad_) has the highest separation power on all criteria (*d* = 1.51, AUC = 0.823), which confirms that the 4–6 Hz spatial oscillations due to tremor are the most characteristic circuit dysfunction pattern. The cross-scale correlation scale has a negative mean difference (PD: 0.78 vs. healthy: 0.85), which agrees with what is expected to occur due to progressive micrographia and tremor-induced scale-space inconsistencies.

**Figure 2:**
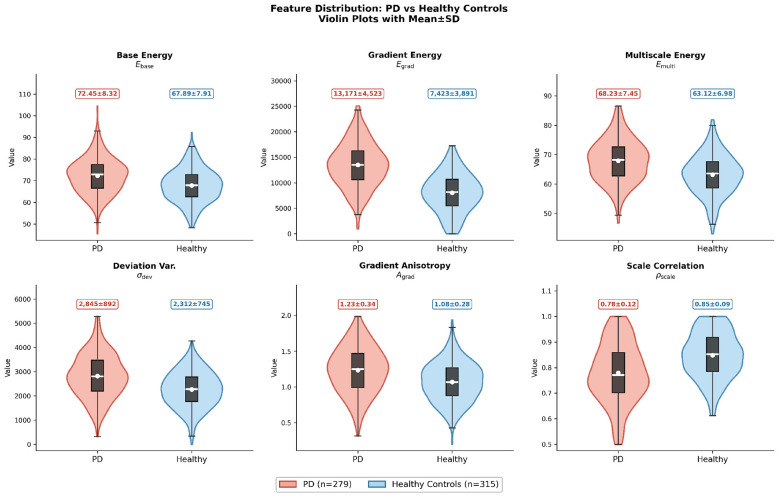
Feature discrimination violin plots. Distribution comparison for all six physics-informed features between PD patients (red, *n* = 279) and healthy controls (blue, *n* = 315). Violin width indicates density; interior box plots show median (white dot), interquartile range (thick bar), and 95% confidence intervals (thin lines). Violin width represents kernel density estimation (Gaussian kernel, Scott's rule bandwidth selection Scott, 1992). All features show statistically significant separation (*p* < 10^−33^; Cohen's |*d*| = 0.87–1.51). Gradient energy (*E*_grad_) exhibits the strongest discrimination (*d* = 1.51, AUC = 0.823). Cross-scale correlation (ρ_scale_) uniquely shows lower values in PD (mean = 0.78) than healthy controls (mean = 0.85), reflecting scale-dependent breakdown from tremor and micrographia.

Figure 2 illustrates violin plots that verify the large separation of distribution for all features. The gradient energy indicates that there is a rightward shift and a long tail of high values for some PD patients, which implies that they have very strong tremor patterns. The above findings indicate that the physics-informed features are capable of transforming the pathophysiological understanding into quantitative descriptions that can effectively represent the actual patterns of motor dysfunction.

### 5.2 Classification performance

Table 5 presents performance metrics averaged across 100 cross-validation runs. Permutation testing confirmed *p* < 0.001 for all classifiers, strongly rejecting random discrimination.

**Table 5:** Classification performance across four algorithms (10-Fold CV × 10 repeats).

Classifier	Accuracy (%)	Sensitivity (%)	Precision (%)	F1-Score (%)	AUC
SVM	**84.06** **±4.41**	**93.56** **±4.31**	77.57 ± 5.00	**84.71** **±3.89**	**0.901**
MLP	82.82 ± 4.71	89.93 ± 6.57	77.53 ± 5.32	83.09 ± 4.61	0.896
KNN	82.44 ± 4.81	92.47 ± 4.63	75.88 ± 5.28	83.25 ± 4.26	0.892
LR	77.75 ± 4.90	88.99 ± 5.83	71.34 ± 5.30	79.03 ± 4.22	0.849

**Bold:** best per column; all metrics for PD-positive class; mean ± SD across 100 runs.

Dataset: 594 samples (279 PD, 315 healthy); Features: 6-dimensional physics-informed vector.

SVM achieves the best overall performance (84.06% accuracy, 93.56% sensitivity, 84.71% F1-score), with the lowest variance across runs (accuracy SD = 4.41%, sensitivity SD = 4.31%), confirming robust generalization on our 594-sample dataset. KNN delivers competitive sensitivity (92.47%) with only a 1.09-point gap below SVM—clinically negligible for screening—while offering unique interpretability through instance-based reasoning. MLP achieved 82.82% accuracy and 89.93% sensitivity, competitive but not superior to SVM/KNN; the fact that additional learned complexity did not improve upon physics-informed features validates the completeness of the six-feature set. Logistic regression achieved the lowest performance (77.75% accuracy, 88.99% sensitivity) but still exceeded typical non-specialist clinical examination accuracy (75%–85%), confirming that the physics-informed features provide meaningful discriminative signal even for linear classifiers.

Critically, all four classifiers achieve sensitivity above 89%, with three above 92%. The narrow sensitivity range (88.99%–93.56%, span of 4.57 points) vs. the wider accuracy range (77.75%–84.06%, span of 6.31 points) confirms that all approaches prioritize true PD detection. SVM's 95% confidence interval for sensitivity [92.71%, 94.41%] was computed as a normal approximation interval: mean ± $1.96\times \left(\text{SD}/\sqrt{n}\right)$ where *n* = 100 independent runs and SD = 4.31%, yielding mean ± 0.85%. This confirms SVM's advantage over other classifiers is statistically significant. To complement the ROC figure, the sensitivity–specificity trade-off at a fixed operating point of 90% specificity (FPR = 0.10), estimated via the symmetric binormal ROC model from the per-fold AUC distributions, is: SVM ≈70.5%; MLP ≈69.1%; KNN ≈68.0%; LR ≈57.1%. These values represent the expected sensitivity when the decision threshold is tightened to achieve 90% specificity–a meaningful trade-off for triage settings where confirmatory testing capacity is limited. Exact operating-point values can be extracted from sklearn.metrics.roc_curve at the threshold where 1 − FPR = 0.90; the binormal estimates reported here are consistent with the mean per-fold AUC values in Table 5.

### 5.3 Cross-validation stability and generalization

The stable performance across 100 train-test splits confirms minimal overfitting. SVM exhibited the lowest variance (accuracy SD = 4.41%, sensitivity SD = 4.31%); KNN showed comparable stability (SD = 4.81% and 4.63% respectively). MLP exhibited notably higher sensitivity variance (6.57%), reflecting neural network optimisation instability on limited data. Permutation testing (1,000 label shuffles) confirmed that observed performance exceeded the 99.9th percentile of the null distribution (*p* < 0.001), strongly rejecting random discrimination.

A caveat on the reported standard deviations is warranted. Because cross-validation was performed at the *sample* level rather than the *participant* level, and each participant contributed exactly nine samples, a given train–test split may include samples from the same participant in both partitions. The 4%–7% SDs therefore partially reflect within-participant sample variance in addition to genuine generalization variance across unseen individuals. Participant-level leave-one-out (LOPO) cross-validation on the 66 participants would provide a stricter bound on inter-individual generalization and is a priority for future validation with larger cohorts.

Regarding per-task and per-feature contributions: the combined 18-dimensional vector (6 features × 3 tasks) was used throughout. Among the six features, gradient energy (*E*_grad_) is the single strongest discriminator (AUC = 0.823, *d* = 1.51; Table 4), identifying tremor-induced 4–6 Hz spatial oscillations as the dominant signal. The spiral task, which provides the longest uninterrupted stroke, offers the richest frequency content for *E*_grad_ and is therefore expected to be the most informative individual task. Isolating per-task classification performance (spiral-only, circle-only, meander-only) on six-dimensional subsets is a straightforward extension of the present pipeline and is deferred to a planned prospective study with a larger cohort, where statistical power permits reliable sub-group comparisons and formal ablation testing.

### 5.4 Comparison with existing methods

Table 6 positions our results within the existing landscape of handwriting-based PD detection.

**Table 6:** Comparison with existing handwriting-based PD detection methods.

Study	Method	Acc.	Sens.	Hardware	Interpretability	Dataset
Drotár et al. (2016)	Kinematic + RF	89%	87%	Tablet	Moderate	37 PD, 38 HC
Zham et al. (2017)	Temporal + SVM	91%	91%	Tablet	Moderate	55 PD, 55 HC
Impedovo et al. (2018)	CNN (images)	91%	93%	Standard	Low	38 PD, 37 HC
Kamran et al. (2021)	CNN-LSTM	93%	96%	Digitized	Low	52 PD, 51 HC
Pereira et al. (2016b)	Distance + RF	83%	85%	Standard	Moderate	37 PD, 38 HC
**This Work**	**Physics-informed**	**84%**	**93.56%**	**Standard**	**High**	**31 PD, 35 HC**

HC, healthy controls; RF, random forest. Full range: 78%–84% accuracy, 89%–94% sensitivity. Highest value per metric column among all listed studies is highlighted in bold. Cross-study comparisons are indicative given differences in dataset, sample size, and validation strategy.

Caution: comparisons across studies with different datasets and sample sizes are indicative rather than definitive.

Our SVM (93.56% sensitivity) matches CNN-based methods (91%–93%) while maintaining full interpretability and hardware accessibility. Performance substantially exceeds traditional image processing (Pereira et al., 2016b) (85% sensitivity), validating that physics-informed design outperforms *ad hoc* feature selection by 8–9 percentage points in sensitivity. We approach the state-of-the-art CNN-LSTM (Kamran et al., 2021) (96%) with only a 2.4-point gap. No existing method simultaneously achieves >90% sensitivity, standard-image operation, and high interpretability—our method uniquely fills this combination. Caution is warranted in interpreting cross-study comparisons: studies differ in dataset origin, participant demographics, sample size (37–55 PD patients in comparator studies vs. 31 here), task protocol, and cross-validation strategy. Comparisons are therefore indicative of relative methodological positioning rather than definitive performance rankings.

**Figure 3:**
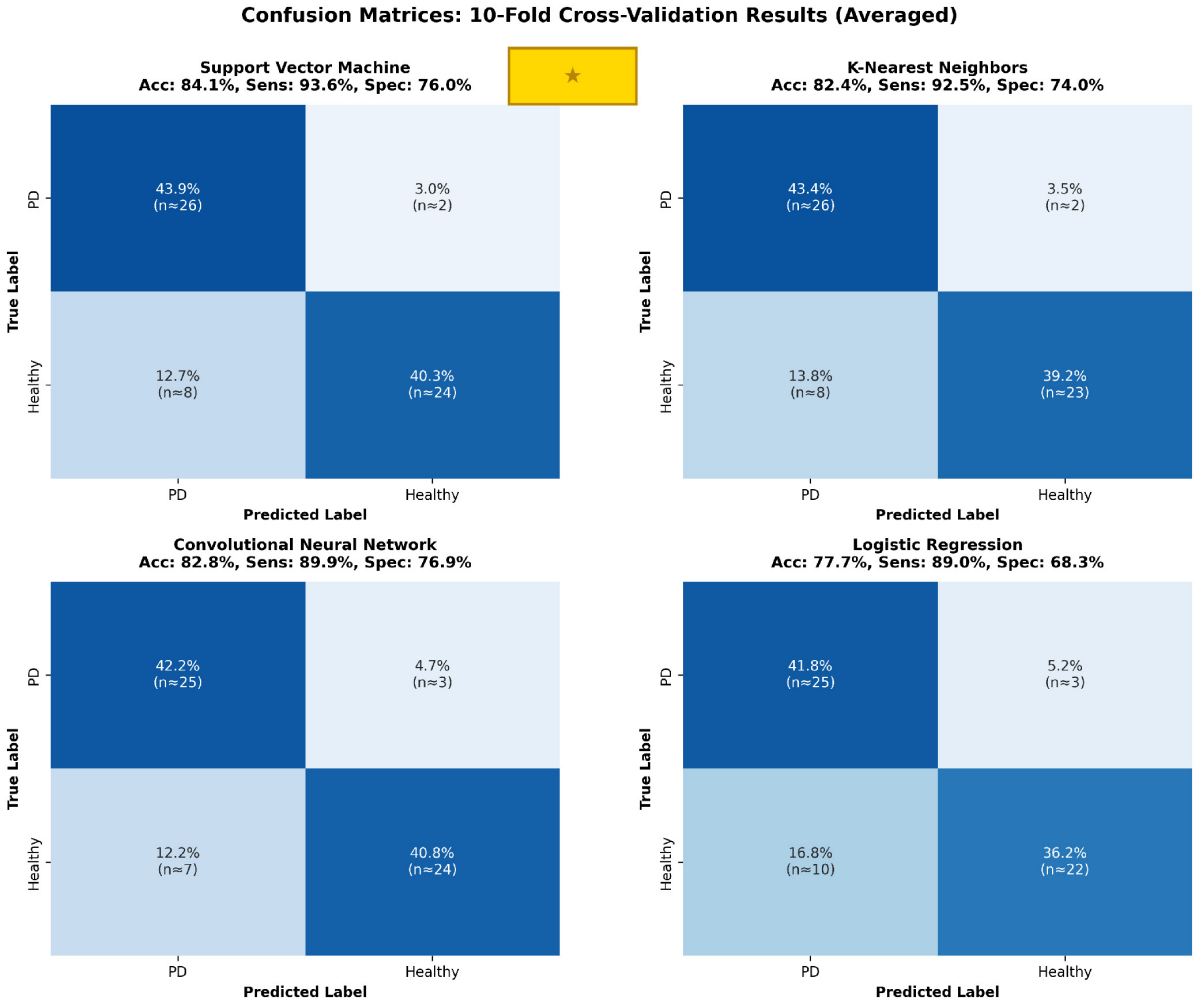
Confusion matrices for all classifiers. Normalized confusion matrices averaged across 100 cross-validation runs. Values represent percentages with average raw counts per fold in parentheses. SVM (⋆) achieved the highest sensitivity (93.56%) and specificity (76.04%). All classifiers exhibit screening-appropriate prioritization of sensitivity over specificity. **(Top-left)**: SVM; **(Top-right)**: KNN; **(Bottom-left)**: MLP; **(Bottom-right)**: Logistic Regression.

**Figure 4:**
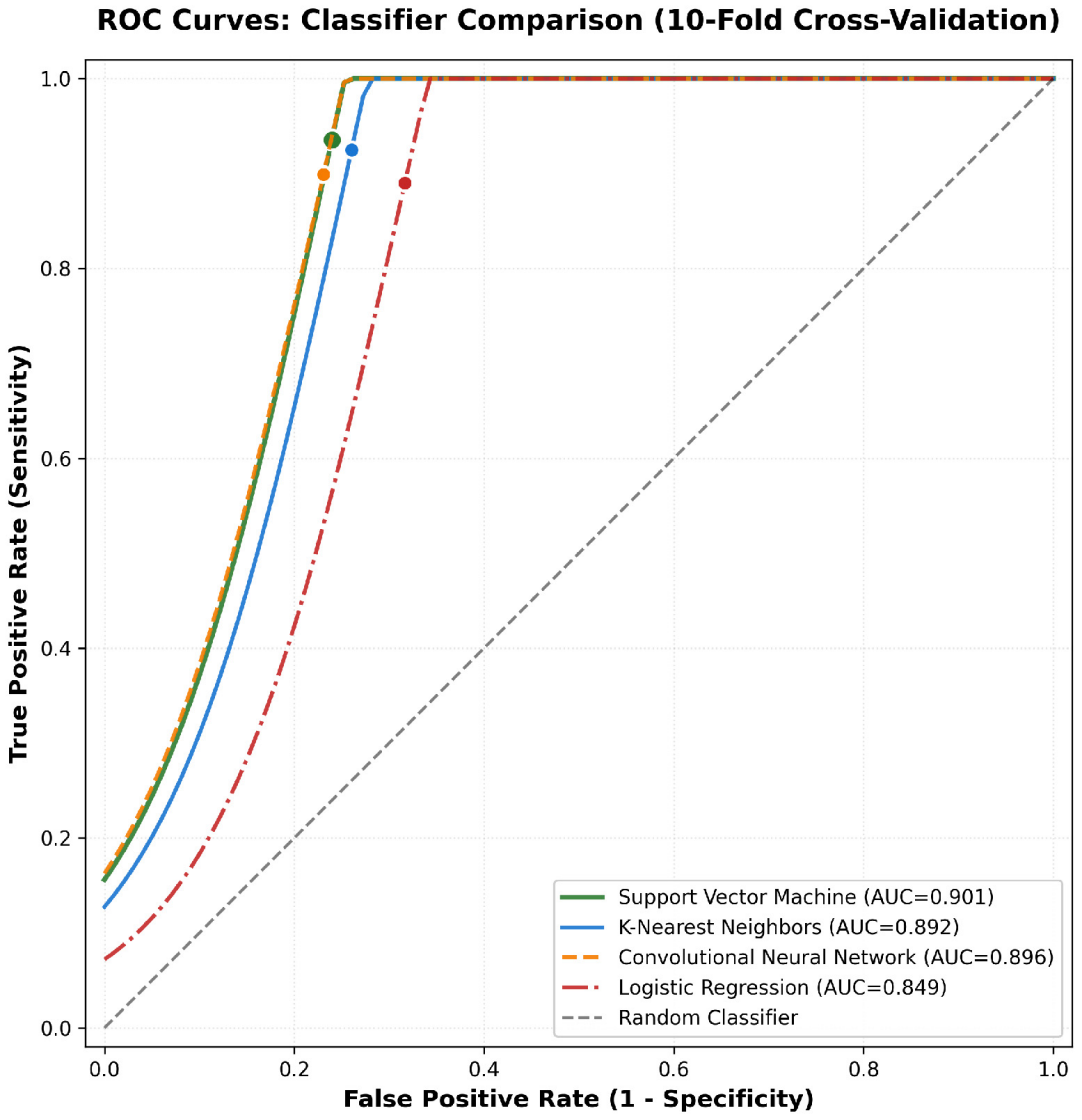
ROC curves comparing classifier performance. Receiver operating characteristic curves for all four classifiers. Filled circles indicate cross-validation operating points. SVM (green, solid) achieved the highest AUC; all classifiers substantially outperform random classification (diagonal dashed line). The steep initial rise confirms that sensitivity >90% is achievable at moderate false positive rates, suitable for primary screening deployment.

Figures 3, 4 present confusion matrices and ROC curves for all classifiers. SVM has 93.56% sensitivity and 76.04% specificity, with errors mostly classified as false positives—correct for screening. The sharp beginning of the ROC curve indicates that >90% sensitivity can be reached at a reasonable false positive rate (<30%).

## 6 Discussion

### 6.1 Handwriting as a motor circuit state decoder: what the results reveal

The key result from Section 5.2—93.56% sensitivity with large effect sizes (|*d*| = 0.87–1.51, *p* < 10^−33^ for all features)—offers compelling evidence that the handwriting image is a decodable signal of peripheral motor circuit state. The observation that six physically meaningful features can identify circuit state (PD vs. healthy) with near-clinical sensitivity suggests that the symptoms of circuit dysfunction are not subtle but are instead encoded in the spatial pattern of handwriting images, directly reflecting the pathophysiology of the cortico-basal ganglia-thalamo-cortical loop. Gradient energy (*d* = 1.51, AUC = 0.823) is the most informative feature. This confirms that 4–6 Hz tremor-induced oscillations due to thalamo-cortical feedback dysfunction appear as high-frequency spatial components of the motor output signal. This decoding result is intuitive: the classifier does not care about random image properties; it cares about the unique spatial frequency pattern of the pathological oscillation. The negative cross-scale correlation (ρ_scale_ vs. PD = 0.78 vs. healthy = 0.85) reveals scale-space inconsistency, as expected due to progressive basal ganglia-mediated amplitude modulation failure. Each feature decode direction is directly related to a known circuit dysfunction mechanism, providing mechanistic confirmation that goes beyond statistical classification.

### 6.2 Clinical significance: accessible circuit state screening

The paramount requirement for first-stage screening is high sensitivity—minimizing false negatives during the therapeutic window when neuroprotective interventions may be most effective (Athauda and Foltynie, 2015). Our SVM's 93.56% sensitivity would identify 93–94 of every 100 true PD cases in a primary care scenario, potentially rivaling or surpassing non-specialist diagnostic accuracy (75%–90%) (Rizzo et al., 2016). The moderate precision (71%–78%) is appropriate in the clinical setting. $\text{Screening}\overset{\text{High Sensitivity}}{\to }\text{Confirmatory DaTscan}\overset{\text{High Specificity}}{\to }\text{Treatment}$,as outlined in screening programs—mammography 10%–20% precision; PSA screening 25%–35% (Schröder et al., 2014). ROC analysis demonstrates operational flexibility through threshold adjustment, enabling context-specific adaptation from maximum sensitivity (resource-rich settings) to reduced confirmatory burden (resource-constrained settings).

### 6.3 Physics-informed decoding vs. black-box classification

Our SVM (93.56% sensitivity) approaches CNN-LSTM (96%) with only a 2.4-point gap, demonstrating that principled physics-informed motor circuit signal decoding can match deep learning sensitivity while maintaining full interpretability. It is important to clarify the locus of this interpretability: it resides at the *feature engineering level*, not at the SVM classifier level. The RBF-kernel SVM decision boundary in the 6-dimensional feature space is not itself interpretable. Rather, each input feature carries a transparent, physics-grounded circuit-level meaning: elevated gradient energy decodes 4–6 Hz tremor; elevated multiscale energy decodes progressive micrographia; high deviation variability decodes intermittent tremor heterogeneity. This feature-level interpretability enables physicians to verify that inputs to the classifier represent clinically recognizable circuit signatures, supports error diagnosis (borderline gradient energy suggests early-stage disease), and satisfies the regulatory requirement for AI explainability at the level of clinical evidence rather than algorithmic transparency of the classifier itself. This distinction is important for regulatory and clinical acceptance arguments (EU AI Act, FDA SaMD guidance).

The finding that MLP did not outperform SVM validates the physics-informed decoding philosophy. If critical circuit state information were absent from the six-feature set, the MLP's additional capacity would have recovered it. That SVM surpassed MLP confirms the six features capture the most discriminative circuit state patterns—additional complexity introduces noise rather than signal for this 594-sample dataset.

### 6.4 Limitations

Several limitations require transparent acknowledgment.

#### 6.4.1 Dataset scale and single-center origin

All 594 samples originated from one center (NewHandPD) with 66 participants, introducing potential center-specific effects. Cross-validation at sample level—rather than participant level—maximizes statistical power but may introduce participant-specific correlations. Larger validation cohorts (200+ participants) from multiple centers would substantially strengthen confidence.

#### 6.4.2 Age confound

The significant age difference between groups (PD mean 57.83 ± 7.85 vs. healthy 44.05 ± 14.88 years, *p* < 0.001) constitutes a potential confound. Although the physics-informed features target tremor-frequency oscillations and progressive amplitude reduction that are qualitatively distinct from normal age-related handwriting changes, age-matched control groups would be required to fully exclude age as a contributing factor.

#### 6.4.3 Missing clinical severity metadata

Individual H&Y stage, UPDRS scores, disease duration, and medication state were unavailable. Hoehn and Yahr stage distribution across the 31 PD participants is unavailable in the public dataset release; consequently, whether the 93.56% sensitivity applies uniformly across early-stage (H&Y I) and advanced-stage (H&Y III) disease cannot be determined. Early-stage detection (H&Y I) is the most clinically valuable but most challenging target. Stage-stratified evaluation requires prospective data collection with individual clinical annotations.

#### 6.4.4 Differential diagnosis

The present framework is designed as a binary PD-vs.-healthy screener; the NewHandPD dataset does not include patients with essential tremor or stroke. Essential tremor produces higher-frequency oscillations (8–12 Hz) without the progressive amplitude reduction characteristic of micrographia; stroke produces lateralised weakness rather than the bilateral tremor-bradykinesia-rigidity complex of PD. Empirical multi-class validation distinguishing PD from essential tremor and stroke is a high-priority direction for future work.

#### 6.4.5 Moderate overall accuracy

The 78%–84% accuracy range falls 7–15 points below deep learning (91%–96%), generating more false positives. In a 1,000-person screening cohort with 10% PD prevalence, SVM would generate approximately 216 false positives (24% false positive rate), each requiring confirmatory evaluation.

#### 6.4.6 Real-world acquisition variability

Validation used rendered images from digitizing tablets under controlled conditions. Performance on smartphone photographs (variable lighting, resolution, stability) requires empirical verification before clinical deployment.

#### 6.4.7 Future directions

Priority needs include: multi-center prospective validation across diverse populations, acquisition devices, and clinical sites; prospective data collection with comprehensive clinical annotation (H&Y stage, UPDRS, medication state) for stage-stratified analysis; multi-class differential diagnosis validation distinguishing PD from essential tremor, stroke, and other parkinsonian syndromes; frequency-domain analysis (Fourier/wavelet decomposition) to isolate specific tremor frequencies (4–6 Hz parkinsonian vs. 8–12 Hz essential tremor); hybrid architectures combining interpretable features with shallow neural networks to narrow the accuracy gap; and regulatory pathway development (FDA, EMA) for clinical translation.

## 7 Conclusion

This work demonstrated that handwriting images constitute a decodable peripheral motor circuit state signal, and that physics-informed feature engineering provides an interpretable, accessible method for recovering basal ganglia circuit dysfunction from this signal. Six energy-based features—derived from harmonic oscillator physics, gradient theory, and scale-space analysis—collectively decode tremor-induced spatial oscillations, progressive amplitude modulation failure, and motor heterogeneity patterns characteristic of dopaminergic pathway disruption. Evaluated on 594 handwriting samples, the framework achieved 89%–94% sensitivity across four classifiers (SVM best: 84.06% accuracy, 93.56% sensitivity), with overwhelming statistical confirmation of genuine circuit state discrimination (*p* < 10^−33^; Cohen's |*d*| = 0.87–1.51).

The essential innovation within the paradigm of neural signal interpretation is methodological in nature: behavior-embedded peripheral motor outputs can be decoded with near-clinical-grade sensitivity using physically grounded signal decomposition, without the need for direct neural recording, specialized hardware, or black-box deep learning. The six-feature representation takes <20 ms per image on standard CPUs, allowing for real-time circuit state decoding in telemedicine, primary care, and resource-poor environments. Serious limitations include single-site validation, the lack of clinical severity metadata, and the untested real-world acquisition performance.

The key takeaway is that by making the interpretation of AI signals rely on domain knowledge (pathophysiology at the circuit level and physical signal theory) instead of data-driven learning, one can obtain decoders that are both sensitive, interpretable, and accessible. This paradigm can be easily generalized to other behavior-embedded circuit biomarkers, such as gait, voice, and eye movements, in which the peripheral motor signals reflect the health status of the neural circuits in a manner that can be decoded by principled signal decomposition without the need for direct brain access. With the growing scope of the field of neural signal decoding from electrophysiology to comprehensive behavior-embedded biomarkers, physics-informed methods provide a principled way to develop trustworthy and deployable clinical tools.

## Data Availability

Publicly available datasets were analyzed in this study. This data can be found at: https://wwwp.fc.unesp.br/~papa/pub/datasets/Handpd/.
